# Basal Linear Deposit: Normal Physiological Ageing or a Defining Lesion of Age-Related Macular Degeneration?

**DOI:** 10.3390/jcm13164611

**Published:** 2024-08-07

**Authors:** Akshaya Lakshmi Thananjeyan, Jennifer Arnold, Mitchell Lee, Cheryl Au, Victoria Pye, Michele C. Madigan, Svetlana Cherepanoff

**Affiliations:** 1 St. Vincent’s Hospital, Victoria Street, Darlinghurst, NSW 2010, Australia; 2 School of Medicine, University of Sydney, Camperdown, NSW 2006, Australia; 3 Marsden Eye Specialists, Parramatta, NSW 2150, Australia; 4 School of Clinical Medicine, University of NSW, Sydney, NSW 2052, Australia; 5 Anatomical Pathology, St Vincent’s Hospital, Darlinghurst, NSW 2010, Australia; 6 School of Medicine, University of Notre Dame, Sydney, NSW 2008, Australia; 7 Optometry and Vision Science, University of New South Wales (UNSW), Sydney, NSW 2052, Australia; 8 Save Sight Institute, University of Sydney, Sydney, NSW 2000, Australia

**Keywords:** age-related macular degeneration, basal linear deposit, basal laminar deposit, ageing

## Abstract

**Objective:** To determine if basal linear deposit (BLinD) is a specific lesion of age-related macular degeneration (AMD). **Methods:** The cohort was selected from a clinically and histopathologically validated archive (Sarks Archive) and consisted of 10 *normal* eyes (age 55–80 years) without any macular basal laminar deposit (BLamD) (Sarks Group I) and 16 *normal aged* eyes (age 57–88 years) with patchy BLamD (Sarks Group II). Only eyes with in vivo fundus assessment and corresponding high-resolution transmission electron microscopy (TEM) micrographs of the macula were included. Semithin sections and fellow-eye paraffin sections were additionally examined. BLinD was defined as a diffuse layer of electron-lucent vesicles external to the retinal pigment epithelium (RPE) basement membrane by TEM and was graded as follows: (i) Grade 0, absence of a continuous layer; (ii) Grade 1, a continuous layer up to three times the thickness of the RPE basement membrane (0.9 µm); (iii) Grade 2, a continuous layer greater than 0.9 µm. Bruch’s membrane (BrM) hyalinisation and RPE abnormalities were determined by light microscopic examination of corresponding semithin and paraffin sections. **Results:** BLinD was identified in both *normal* (30%) and *normal aged* (62.5%) eyes. BLinD was thicker in *normal aged* eyes (*p* = 0.045; 95% CI 0.04–3.4). BLinD thickness positively correlated with both the degree of BrM hyalinisation (*p* = 0.049; 95% CI 0.05–2.69) and increasing microscopic RPE abnormalities (*p* = 0.022; 95% CI 0.188–2.422). RPE abnormalities were more likely to be observed in eyes with increased BrM hyalinisation (*p* = 0.044; 95% CI 0.61–4.319). **Conclusions:** BLinD is most likely an age-related deposit rather than a specific lesion of AMD. Its accumulation is associated with increasing BrM hyalinisation and microscopic RPE abnormalities, suggesting a relationship with dysregulated RPE metabolism and/or transport.

## 1. Introduction

Age-related macular degeneration (AMD) is a clinically diagnosed disease. Upon fundus ophthalmoscopy, early and intermediate AMD is defined by the presence of soft drusen, with or without pigment changes, whereas late AMD is defined by the presence of geographic atrophy and/or macular neovascularisation (advanced AMD) [1]. Sarks [2] was the first to document the continuum of specific histological changes that accompany each stage of AMD in a large cohort of clinically validated eyes. A continuous and diffuse layer of sub-retinal pigment epithelium (RPE) basal laminar deposit (BLamD) was shown to be the defining histological lesion of AMD. A second diffuse deposit, basal linear deposit (BLinD), was later revealed by transmission electron microscopy (TEM) to be present in all eyes with AMD [3]. BLinD appears as electron-lucent vesicles that accumulate in the inner collagenous zone (ICZ) of Bruch’s membrane (BrM), external to the RPE basement membrane. Because BLinD is seen in continuity with soft drusen [4] and is composed of similar electron-lucent vesicles, it is considered a soft drusen precursor [5].

There is extensive evidence that the lipid content within BrM increases with age [6]. There is also substantial evidence that the electron-lucent vesicles that comprise BLinD and soft drusen are lipid-rich [7,8]. Subsequent histological and ultrastructural investigations showed that a BLinD-like deposit could also be found in younger eyes without AMD [4]. These observations raise an important question: is BLinD a specific AMD lesion or is it associated with normal ageing?

One of the difficulties in reconciling published findings to date is the lack of a universal definition of BLinD. The study cohorts on which observations have been based were mixed, with mostly small sample sizes and without in vivo clinical validation. This limits the clinical and histological correlation of identified markers. To address this question, we studied a clinically and histologically validated archive of 10 normal and 16 normal aged human eyes. Using a standardised definition of BLinD, we measured its thickness in electron micrographs and correlated our findings to age as well as light microscopic evaluation of BrM hyalinisation and RPE abnormalities.

## 2. Materials and Methods

### 2.1. Ethics

The study protocol was approved by the St Vincent’s Hospital Human Research Ethics Committee (REF: 2021/ETH01147) and was monitored by the St Vincent’s Hospital Sydney Research Office. Archival records were accessed for research from January to December 2023. Authors did not have access to sufficient information to identify participants during or after data collection.

### 2.2. Cohort

The Sarks archive consists of over 600 eyes obtained from 1967 to 1995 from over 350 prospectively consenting patients, each with detailed clinical annotations and histological assessment. During the clinical assessment, the best corrected distance visual acuity, fundus assessment, medical history and other intra-ocular findings [2] were documented. In vivo fundus photography was captured when possible. *Post-mortem*, macular histology was assessed by light microscopy. TEM assessment was also performed in most cases on fellow eyes. In vivo fundus findings and clinical progression were correlated with detailed histological and ultrastructural analyses.

### 2.3. Sarks Histological AMD Grading

Sarks [2] was the first to define ageing and AMD as a continuum of histological changes in a large cohort of clinically validated human eyes. Group I (normal) eyes have no macular BLamD and have a normal fundus. Group II (normal aged) eyes contain patchy, macular BLamD. Group III (early AMD) eyes have a continuous (at least 250 µm), thin (less than half the height of an RPE cell) layer of macular BLamD. The presence of continuous BLamD marks the histological threshold of AMD. Group IV (intermediate AMD) eyes are characterised by continuous, thick (greater than half the height of an RPE cell) macular BLamD, corresponding to the presence of early and intermediate AMD upon fundus examination. Group V eyes are defined by the presence of geographic atrophy and Group VI by disciform scar formation.

### 2.4. Cohort Inclusion and Exclusion Criteria

Eyes from the Sarks Archive were selected if they met the following inclusion criteria: (i) Group I (normal; no macular BLamD) and Group II (normal aged; patchy macular BLamD); (ii) at least two TEM images of the macula available for assessment; (iii) corresponding macular resin semithin sections for light microscopic evaluation and (iv) well-documented in vivo fundus findings. Cases with paraffin sections of the fellow eye were prioritised for inclusion. Eyes with any macular pathology were excluded. Normal clinical fundus examination findings were confirmed by a review of the clinical notes and, where available, in vivo fundus photographs. Light microscopic review of semithin resin sections and picro-Mallory-stained paraffin sections was undertaken to confirm (i) the absence of AMD, defined by the absence of continuous macular BLamD and (ii) the absence of any other microscopic macular pathology.

### 2.5. Light and Electron Microscopy

Eyes prepared for TEM were fixed in 2.5% glutaraldehyde in 0.1 M cacodylate buffer, and the macula and optic disc were punched out using an 8 mm corneal trephine [9,10]. After the retina and choroid were dissected away, the tissue was subdivided into blocks and washed in fresh cacodylate buffer, then postfixed in 2% osmium tetroxide in 0.1 M cacodylate buffer. This was followed by 2% uranyl acetate staining. Next, tissue blocks were dehydrated through graded alcohols and acetone, before being embedded in Spurrs low-viscosity resin and cured at 60 °C for 8 h. Semithin 0.5 µm sections were cut and stained with toluidine blue. Ultrastructural areas of interest were identified on semithin resin sections and examined by TEM (Philips 300 Electron Microscope; Eindhoven, The Netherlands) at an accelerating voltage of 80 Kv. TEM micrographs were examined after digital scanning.

Paraffin section methods, described previously [2], were as follows: after fixation in neutral buffered formalin and paraffin embedding, the pupil-to-optic nerve block was sectioned at 8 µm thickness through the macula. Every 10th paraffin section was stained with picro-Mallory trichrome. Coverslipped glass slides of resin and paraffin sections were digitally scanned for microscopic review and data collection.

### 2.6. Bruch Membrane Hyalinisation

BrM hyalinisation was graded using a scale previously defined by Sarks [2] and later validated by van der Schaft et al. [11]. Briefly, Grade 1 hyalinisation does not extend beyond BrM; Grade 2 hyalinisation reaches the intercapillary pillars; Grade 3 hyalinisation extends through the intercapillary pillars entirely and Grade 4 hyalinisation encircles the choriocapillaries.

### 2.7. Basal Linear Deposit: Definition

The term “basal linear deposit” was originally used to describe what is now understood to be BLamD, a diffuse, aberrant basement membrane-like deposit *internal* to the RPE basement membrane that is readily identifiable upon *light microscopic* examination of *paraffin sections* after histological staining [2,12]. The distinct properties of BLamD and BLinD only became apparent with the routine availability of TEM. BLinD was first defined in published literature as a layer of electron-lucent vesicles external to the RPE basement membrane using TEM [13]. It was further defined as the precursor lesion for soft drusen in 1994 [14] and subsequently confirmed to be present in all eyes, with histological evidence of early AMD defined as a continuous layer of macular BLamD [3]. There are no published criteria for defining BLinD in terms of thickness or extent in tissue cross-sections. In the absence of published consensus criteria, we defined BLinD as a diffuse layer of electron-lucent vesicles external to the RPE basement membrane that is at least the thickness of the RPE basement membrane (approximately 0.3 µm) [15]. Here, we use the term “electron-lucent vesicles” purely as an ultrastructural morphological descriptor without any functional or biochemical inferences. This deposit was classified as BLinD only when it was seen spanning the entirety of a TEM photograph at low magnification (13.5 × 1000) (approximately 9 µm). The maximum thickness of BLinD in each eye was recorded and graded into the following categories: (i) less than 0.3 µm (Grade 0); (ii) up to 3 × 0.3 µm (Grade 1); and (iii) greater than 3 × 0.3 µm (Grade 2). Three other histological landmarks were used to confirm BLinD thickness: (i) the diameter of a red blood cell within the choriocapillaris, approximately 8 µm [16]; (ii) the diameter of a macular RPE nucleus, approximately 8–12 µm [17]; and (iii) the height of a macular RPE cell, approximately 14 µm [18].

### 2.8. Imaging and Scanning

Coverslipped glass slides of stained paraffin and resin semithin sections were scanned using an Olympus Slideview VS200 (Olympus Life Science; Tokyo, Japan), a high throughput brightfield and fluorescent slide scanner. The 60×/1.42 oil objective and brightfield imaging mode were used for scanning all slides. Once scanned, slides were assessed using the OlyVIA software Version 2.9.1 (Olympus Life Science; Tokyo, Japan). Kodachromes and archival paper photographs were scanned using the Epson Perfection V700 Photo Scanner (Epson Australia Pty Ltd., North Ryde, Australia).

### 2.9. Statistical Analysis

Statistical analysis was performed using GraphPad Prism 8 (GraphPad Software, San Diego, CA, USA) and SPSS Version 28 (IBM Corp, Armonk, NY, USA). The Fisher exact test was used to analyse (i) the presence of BLinD vs. Sarks Group I/II; (ii) RPE detachment on paraffin sections vs. the presence of BLinD upon TEM; and (iii) the presence of a sub-RPE space on semithin resin sections vs. the presence of BLinD upon TEM. A two-group *t*-test was used to evaluate the mean age and the mean number of TEM and semithin sections analysed between Sarks Groups I and II. Ordinal regression was used to analyse (i) BLinD grade vs. Group I/II; (ii) BrM hyalinisation grade vs. Group I/II; (iii) BrM hyalinisation vs. BLinD grade; (iv) BrM hyalinisation vs. age; (v) RPE microscopic morphology vs. Group I/II; and (vi) RPE morphology vs. BrM hyalinisation.

## 3. Results

### 3.1. Cohort

**Figure 1:**
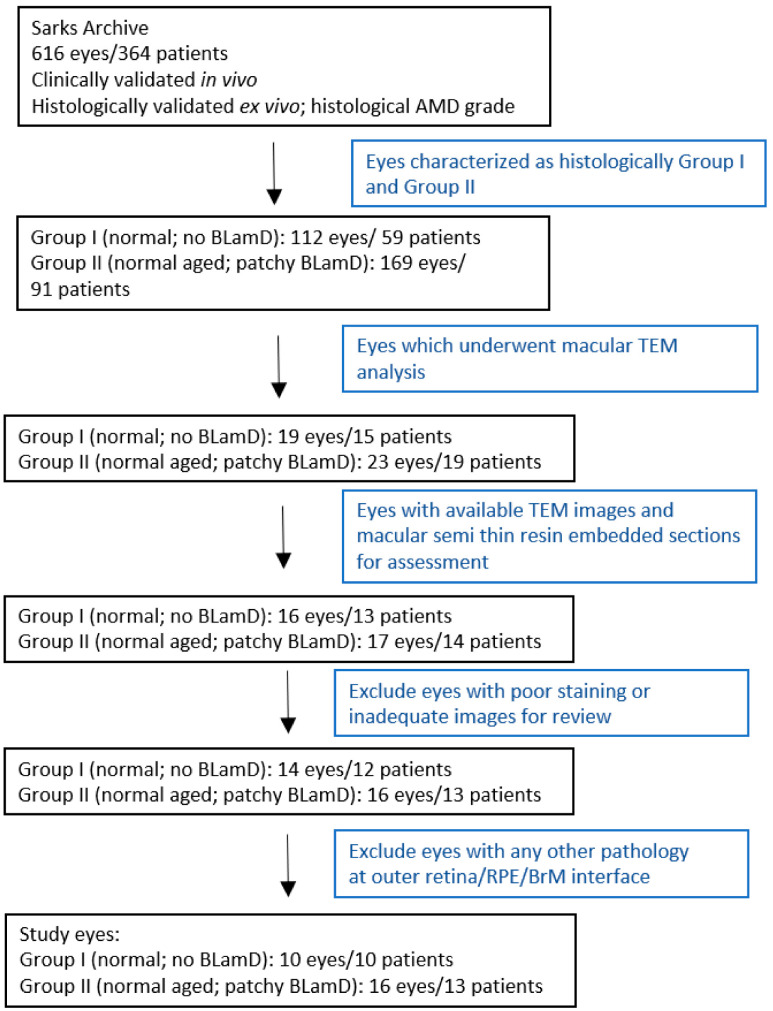
Study cohort: selection criteria. Abbreviations: BLamD—basal laminar deposit; TEM—transmission electron microscopy; RPE—retinal pigment epithelium; BrM—Bruch’s membrane.

A total of 10 Sarks Group I (mean age 63.0 ± 8.3 years) and 16 Group II eyes (mean age 74.2 ± 7.3 years) (Figure 1) met the inclusion criteria summarised in Table 1. Group I eyes were significantly younger (*p* = 0.0014, 95% CI 4.8–17.6), with a trend towards better visual acuity (*p* = 0.11, 95% CI –0.05–0.5), compared with Group II. The interval between death and tissue fixation ranged from 1 to 12 h. The mean time from the last clinical review and patient death was 12.6 months in Group I and 18.1 months in Group II. There was no statistical difference in the number of semithin sections examined in Group I compared to Group II (7.5 ± 6.2 vs. 5.4 ± 3.8; *p* = 0.30, 95% CI –6.1–1.9) or in the number of TEM photographs reviewed per eye between the two groups (25.6 ± 20.4 vs. 19.0 ± 14.6; *p* = 0.34, 95% CI –20.7–7.53).

### 3.2. Basal Linear Deposit in Normal and Normal Aged Eyes

**Figure 2:**
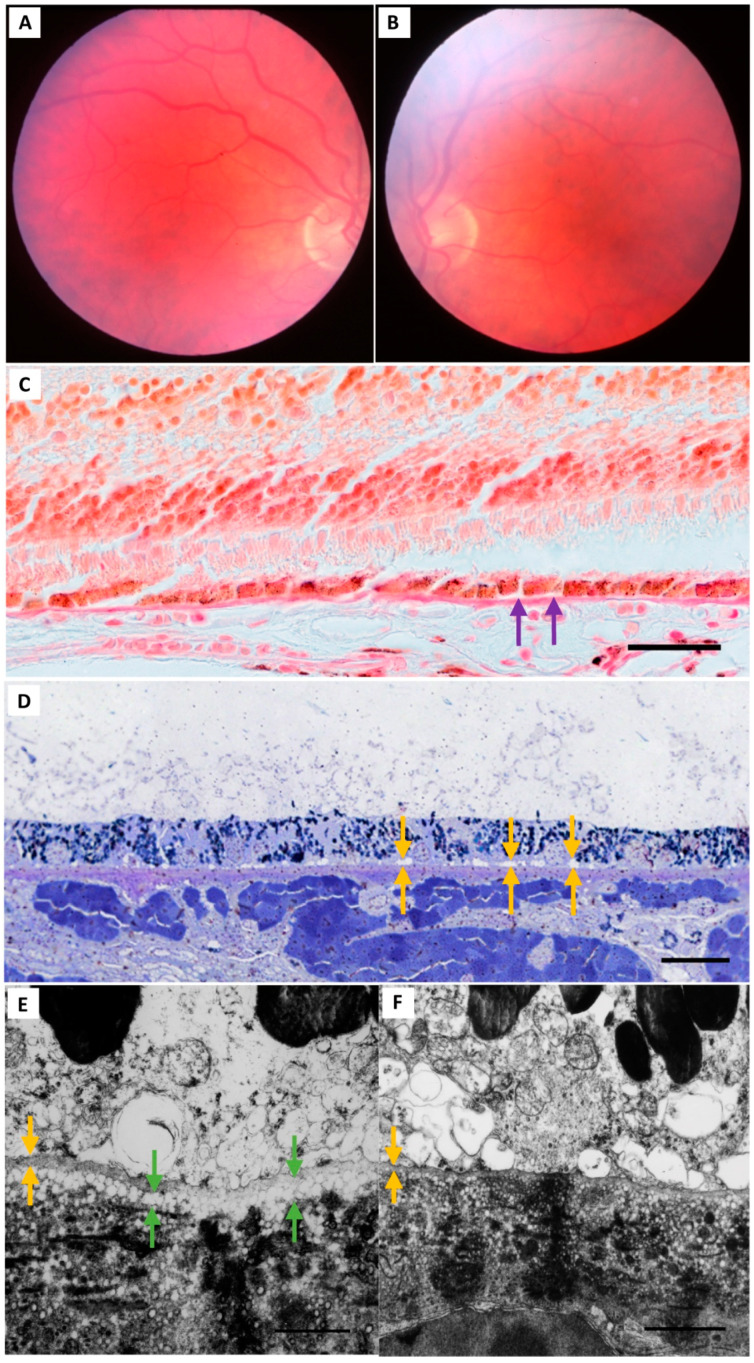
Study cohort: Group I (normal) eyes. epresentative Group I eye (55 year old male) with a normal bilateral fundus examination at the last clinical visit (**A**,**B**). Macular sections were examined under light (left and right eyes) and electron (right eye) microscopy. (**C**) Foci of RPE separation from BrM (purple arrows) are visible on the paraffin section of the macula. (**D**) In the semithin resin section, a small, unstained space is just visible between the base of the RPE and BrM (yellow arrows). (**E**) This space consists of a layer of vesicles when viewed by TEM (green arrows) and is external to the RPE basement membrane (yellow arrow). There is some variability in the thickness of this layer across different ultrastructural fields (**F**), seen here at 173,600× magnification. Scale bars: C = 20 µm; D = 20 µm; E = 1 µm; F = 1 µm. Paraffin section: picro-Mallory trichrome stain. Abbreviations: RPE—retinal pigment epithelium; BrM—Bruch’s membrane; TEM—transmission electron microscopy.

**Figure 3:**
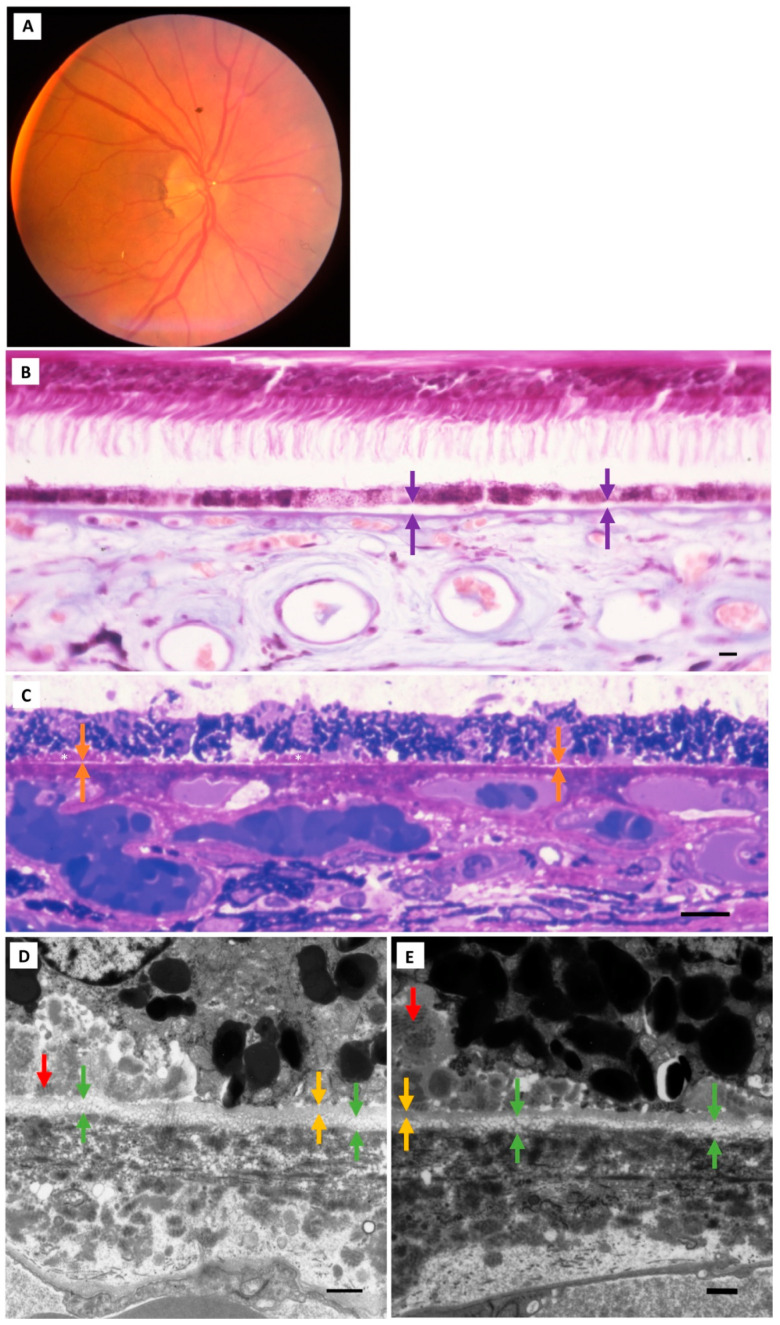
**Study cohort: Group II (normal aged) eyes**. (**A**) Representative Group II eye (85-year-old male). The fundus photo of the right eye at the last examination is shown. Macular sections were examined under light (left and right eyes) and electron (right eye) microscopy. (**B**) The macular paraffin section of the right eye shows diffuse RPE detachment (purple arrows) from BrM (Grade 3 hyalinisation). (**C**) In the semithin section, there are focal separations of the RPE (orange arrows) from the hyalinised BrM. Early-type BLamD is seen in thin patches (between asterisks). (**D**,**E**) In the TEM micrograph, BLinD (green arrows) is seen external to the RPE basement membrane (yellow arrows). Patchy BLamD (red arrows) is seen internal to the RPE basement membrane. Paraffin section scale bar = 15 µm; picro-Mallory trichrome stain. Resin section scale bar = 15 µm; toluidine blue stain. TEM scale bar: 1 µm. Abbreviations: RPE—retinal pigment epithelium; BLamD—basal laminar deposit; BLinD—basal linear deposit; TEM—transmission electron microscopy.

**Figure 4:**
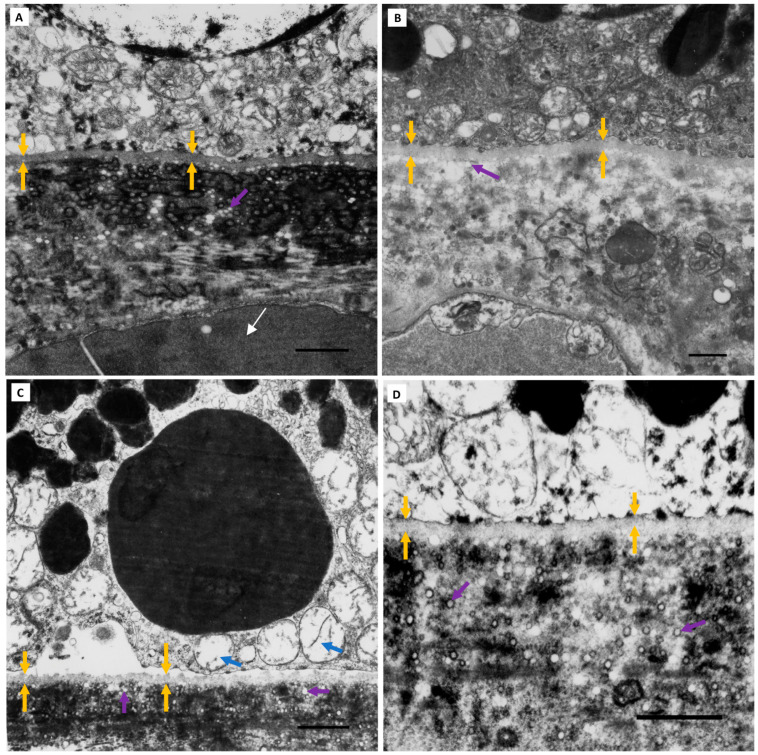
**Grade 0 BLinD.** Electron-lucent vesicles (purple arrows) external to the RPE basement membrane (yellow arrows) were present on the TEM micrographs in all study eyes, even those not meeting our definition for BLinD (a diffuse layer at least as thick as the RPE basement membrane). (**A**,**B**) TEM micrographs of two representative Group I (normal) eyes. Part of a choriocapillary red blood cell (white arrow) is captured. (**C**,**D**) Two representative Group II (normal aged) eyes with absent BLinD. Eyes without BLinD were almost twice as frequent in Group I (7/10; 70%) compared with Group II (6/16; 37.5%). The large, heterogenous, electron-dense structure next to mitochondria (blue arrow) in C likely represents melanolipofuscin. Scale bar: 1 µm. Abbreviations: RPE—retinal pigment epithelium; TEM—transmission electron microscopy; BLinD—basal linear deposit.

**Figure 5:**
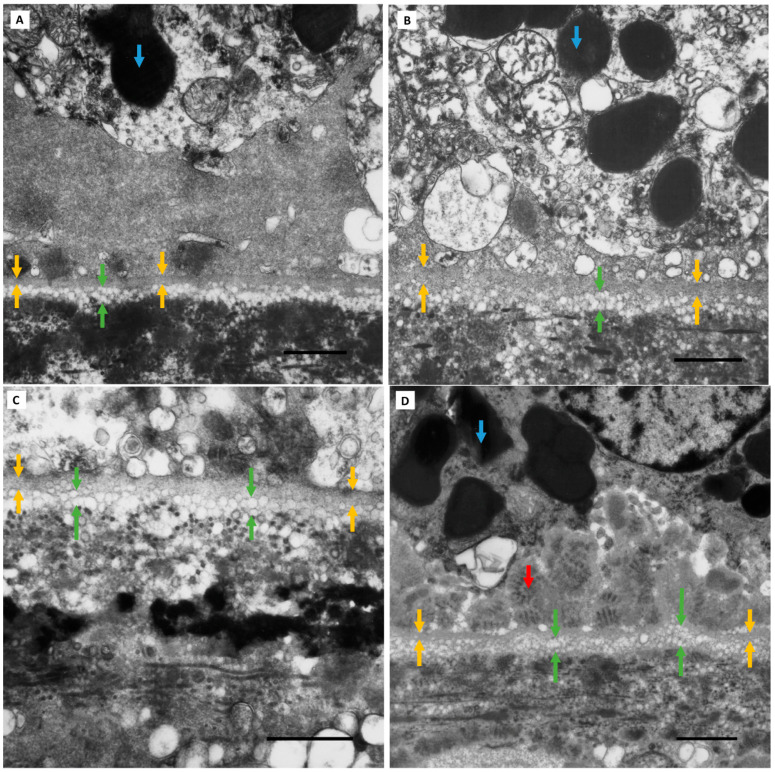
**Grade 1 BLinD.** Grade 1 BLinD (green arrows) was defined as up to 3 times the thickness of the RPE basement membrane (yellow arrows): 3 × 0.3 µm = approximately 0.9 µm was found in 2/10 Group I (normal) eyes and 3/16 Group II (normal aged) eyes. Macular TEM micrographs of Group I (**A**,**B**) and Group II eyes (**C**,**D**). Note the vesicles seen traversing the inner collagenous, elastic and outer collagenous layers of BrM, which become smaller and more dispersed with increasing distance from the RPE basement membrane. The larger vacuoles seen in (**C**,**D**) are within the cytoplasm of choriocapillary endothelial cells (best illustrated in (**D**)). Pigment granules (blue arrows) within the basal RPE cytoplasm are seen admixed with mitochondria. A patch of early-type BLamD, composed largely of long-spacing collagen (red arrow) is captured in (**D**). Scale bar: 1 µm. Abbreviations: BLinD—basal linear deposit; RPE—retinal pigment epithelium; TEM—transmission electron microscopy; BLamD—basal laminar deposit; BrM—Bruch’s membrane.

**Figure 6:**
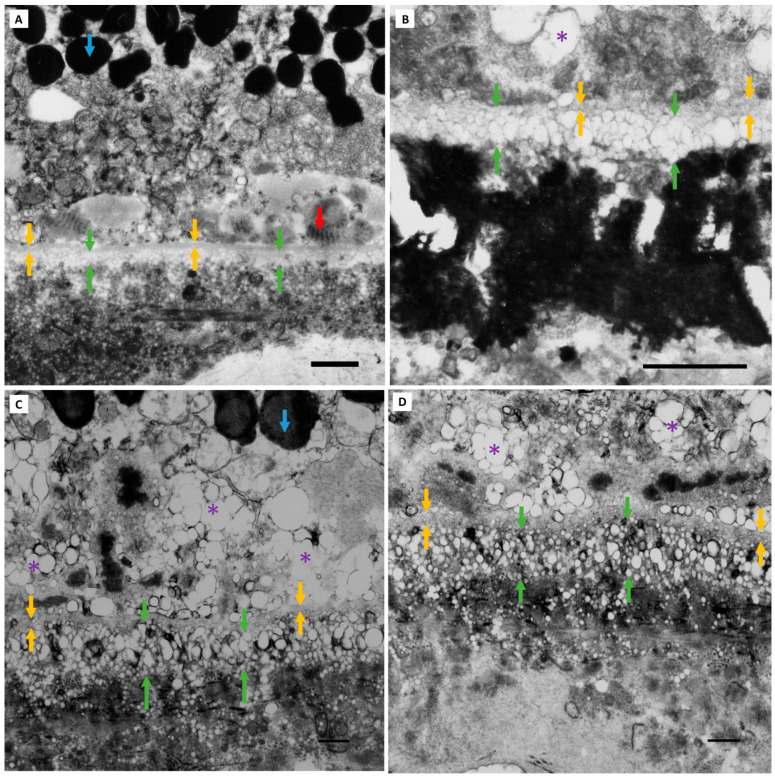
**Grade 2 BLinD.** Grade 2 BLinD (green arrows), defined as more than 3 times the thickness of the RPE basement membrane (>3 × 0.3 µm ≥ 0.9 µm) (yellow arrows), was only seen in Group II (normal aged) eyes (7/16). Macular TEM micrographs of 4 representative Group II eyes are shown in (**A**–**D**). The blue arrows identify RPE cytoplasmic pigment granules. A patch of early-type BLamD (red arrow) is captured in (**A**). Again, electron-lucent vesicles are seen extending to the outer collagenous zone of BrM, decreasing in size and becoming sparser with increasing distance from the RPE basement membrane. Of note, larger lipid vesicles are seen internal to the RPE basement membrane, within the basal RPE cytoplasm (purple asterisk), best demonstrated in (**C**,**D**). TEM scale bar: 1 µm. Abbreviations: BLinD—basal linear deposit; RPE—retinal pigment epithelium; TEM—transmission electron microscopy; BLamD—basal laminar deposit; BrM—Bruch’s membrane.

Electron-lucent vesicles were found in BrM in all study eyes. These were seen traversing through the RPE basement membrane and dispersed throughout all layers of BrM, from the inner to the outer collagenous zones. The vesicle size generally decreased with increasing distance from the RPE basement membrane as seen by TEM (Figure 2, Figure 3, Figure 4, Figure 5 and Figure 6). A diffuse layer meeting our definition for BLinD was found in both normal (Group I) and normal aged (Group II) eyes (Table 2). Representative in vivo fundus photos and the corresponding macula paraffin, resin and TEM findings are shown in Figure 2 and Figure 3. BLinD thickness grades are shown in Figure 4, Figure 5 and Figure 6. BLinD was more frequently observed in Group II (10/16; 62.5%) compared with Group I (3/10; 30%) eyes. While this difference was not statistically significant (Fisher’s exact test *p* = 0.23), the thickness of BLinD was greater in Group II (up to Grade 3) compared with Group I (up to Grade 2) (ordinal regression *p* = 0.045; 95% CI 0.04–3.4).

### 3.3. Bruch’s Membrane Hyalinisation, RPE Abnormalities and Basal Linear Deposit

BrM was less hyalinised in Group I eyes (Grades 0 to 2) compared with Group II (Grades 1 to 3) (ordinal regression: *p* = 0.013; 95% CI 0.6–5.3). Increasing BrM hyalinisation was associated with increasing age across both groups (ordinal regression: *p* < 0.08, 95% CI 0.038–0.253) (Table 2). There was a positive correlation between BLinD thickness and the degree of BrM hyalinisation (ordinal regression *p* = 0.049; 95% CI 0.05–2.69). The morphology of the macular RPE was largely preserved and seen as a cuboidal monolayer in both groups, although mild abnormalities (focal decrease or increase in pigmentation, mild attenuation or hypertrophy) were more frequently observed in Group II (*p* = 0.06; 95% CI −0.89 to 4.484) (Table 2). Mild RPE abnormalities were positively correlated with both BrM hyalinisation (*p* = 0.044; 95% CI 0.61–4.319) and BLinD thickness (*p* = 0.022; 95% CI 0.188–2.422).

### 3.4. BLinD in AMD Eyes

**Figure 7:**
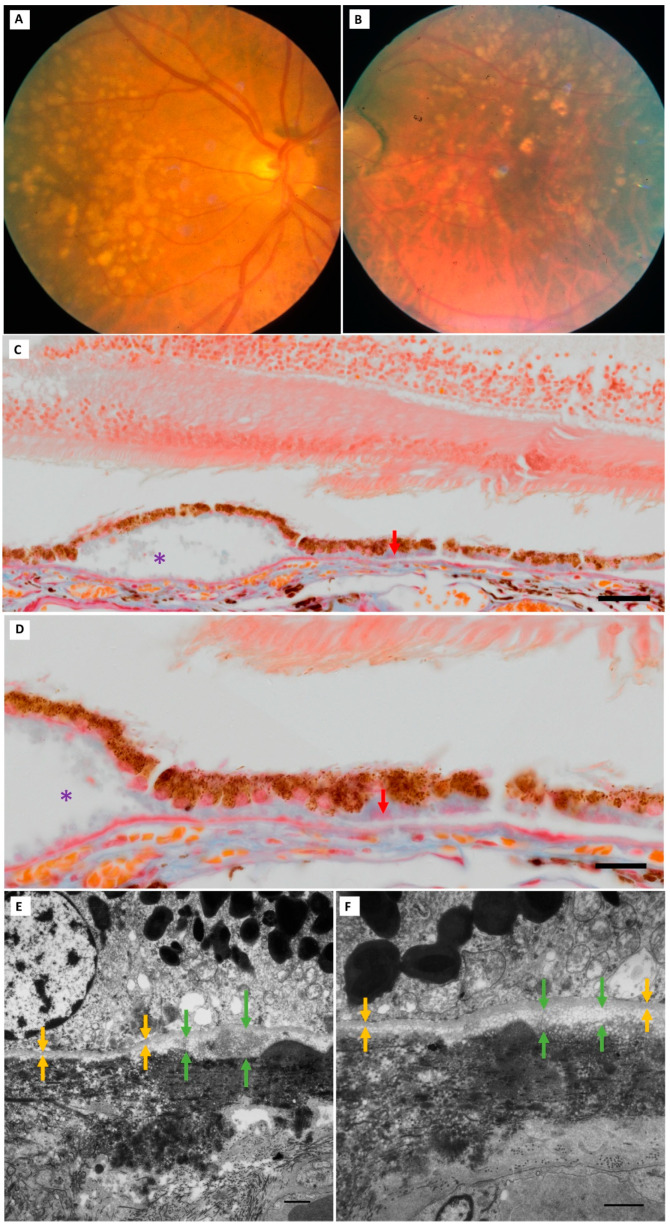
**BLinD thickness in clinically and histologically validated AMD eyes.** (**A**,**B**) A Sarks Group IV (intermediate AMD) eye (75-year-old male). Bilateral soft drusen and pigment changes at the last in vivo fundus examination. (**C**,**D**) Thick, continuous BLamD (red arrows) of both early (pale blue staining) and late (magenta inclusions) types is seen in the paraffin section of the left eye. Soft drusen appear as “optically empty” dome-shaped separations of both the RPE and BLamD from the hyalinised BrM (purple asterisk). RPE abnormalities are conspicuous, consisting of hypertrophy or attenuation and hyper- and hypopigmentation. Variability in BLinD thickness (green arrows) beneath the RPE-BM (yellow arrows) is seen in the right eye, shown here in two TEM fields (**E**,**F**) without soft drusen. Paraffin section scale: C = 50 µm, D = 20 µm. TEM scale bar: 1 µm. Abbreviations: BLinD—basal linear deposit; RPE—retinal pigment epithelium; TEM—transmission electron microscopy; BLamD—basal laminar deposit; BrM—Bruch’s membrane.

Variations in BLinD thickness were observed between eyes in both Group I and II eyes and were also seen within the same eye across different TEM fields. To determine if this was also true for AMD eyes, we examined a representative clinically and histologically validated eye from Sarks Group III (early AMD) and Group IV (intermediate AMD). BLinD thickness also varied within both eyes (Figure 7). Group III and IV eyes also had a greater degree of RPE detachment in semithin resin sections and more conspicuous RPE abnormalities by light microscopy.

### 3.5. RPE Detachment and BLinD

**Figure 8:**
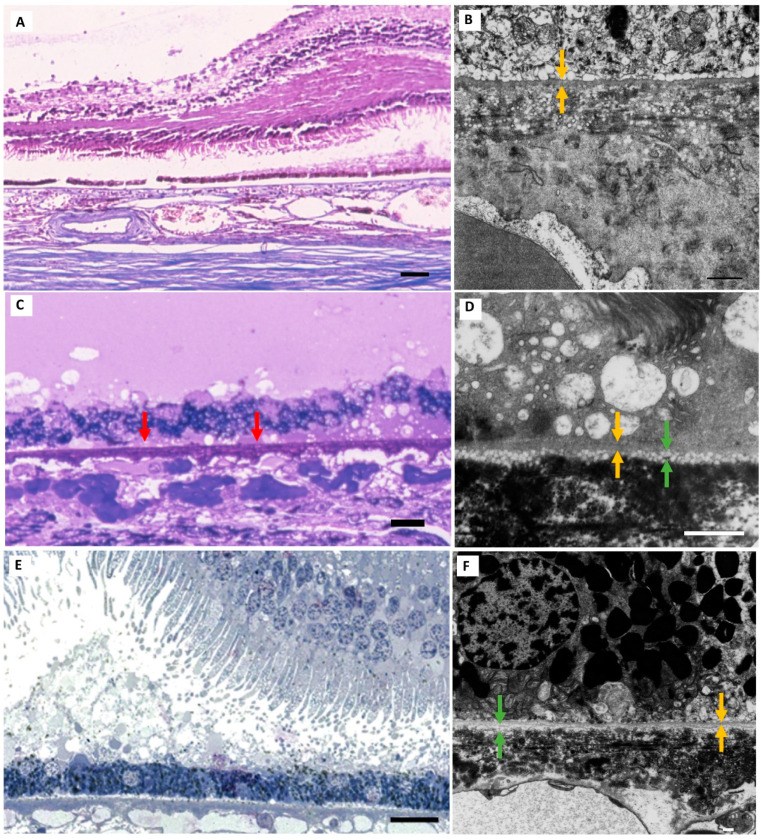
**Separation of the RPE from BrM**. RPE separation from BrM was not a reliable light microscopic marker for the presence of BLinD in this cohort of normal and normal aged eyes. A representative Group I eye with diffuse RPE detachment from BrM on paraffin section (**A**) and Grade 0 BLinD using TEM (**B**); note, instead, a layer of larger vesicles internal to the RPE basement membrane (yellow arrows). Areas of patchy BLamD (red arrows) and no RPE separation is seen in this macular semithin section (**C**) from a representative Group II eye with grade 1 BLinD (green arrows) by TEM (**D**). Diffuse separation of the RPE from BrM in the macular semithin section of a Group I eye (**E**) with grade 1 BLinD by TEM (**F**). TEM scale bar: 1 µm. Resin section scale bar: 15 µm. Paraffin section scale bar: 75 µm. Abbreviations: BLinD—basal linear deposit; RPE—retinal pigment epithelium; TEM—transmission electron microscopy; BLamD—basal laminar deposit; BrM—Bruch’s membrane.

To determine if separation of the RPE from BrM on paraffin and resin sections could be used as a light microscopic marker of BLinD, we correlated this finding to the presence of BLinD upon TEM. RPE separation from BrM was more frequently seen in paraffin sections compared with resin sections (Table 2), and the degree of RPE separation was greater. Diffuse RPE separation was only seen in paraffin sections (Figure 8A). There was no significant association between the presence of BLinD by TEM and RPE separation in paraffin (Fisher’s exact test *p* > 0.99) or resin (*p* = 0.12 for resin) sections. BLinD was observed in eyes with and without RPE separation in both paraffin and semithin sections (Figure 8). There was no association between the RPE separation observed in paraffin sections and the RPE separation observed in resin sections (Fisher’s exact *p* > 0.99).

### 3.6. Diffuse Vesicle Layer Internal to the RPE Basement Membrane

**Figure 9:**
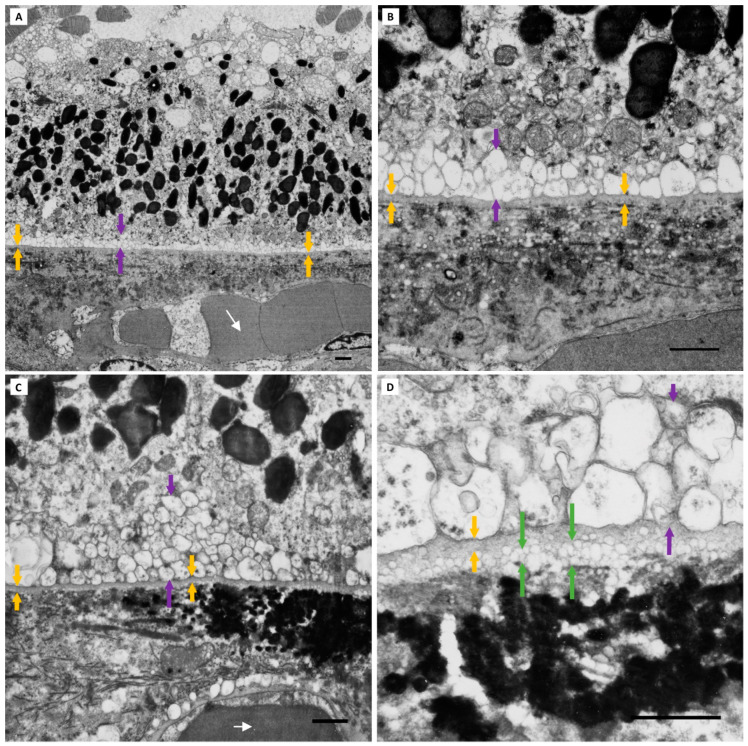
**Diffuse layer of vesicles internal to the RPE basement membrane.** A diffuse layer of larger, electron-lucent vesicles was seen in 3 of the 26 eyes in the normal and normal aged cohort. (**A**,**B**) A Group I eye with Grade 0 BLinD and a diffuse layer of large-vesicle deposition (purple arrows) internal to the RPE basement membrane (yellow arrows). (**C**,**D**). The same layer seen in another eye with Grade 1 BLinD (green arrows). Note the smaller electron-lucent vesicles traversing the RPE basement membrane (**D**) that extends into the inner collagenous zone through the elastin layer and into the outer collagenous zone of BrM (**B**), where some can be seen coalescing around the wall (**C**) of a choriocapillary. White arrows identify red blood cells in the choriocapillary lumen. TEM scale bar: 1 µm. Abbreviations: BLinD—basal linear deposit; RPE—retinal pigment epithelium; TEM—transmission electron microscopy.

In 3 of 26 normal and normal aged eyes, we observed a diffuse layer of electron-lucent vesicles internal to the RPE-BM by TEM (Figure 9). These vesicles were larger in size than those found within BLinD and often at least half the size of an RPE mitochondrion (at least 0.5 um). This layer has not been previously described in the published literature and was observed in two eyes with BLinD (n = 1 Group I; n = 1 Group II) and one eye without BLinD (Group I). There was no RPE detachment in the semithin sections for two of these eyes and focal detachment in one eye. Paraffin sections were available for one eye and showed diffuse RPE detachment. Identifying this layer prompted a review of all TEM micrographs of the study cohort. Similarly large, electron-lucent vesicles were seen in Group I and II eyes, irrespective of BLinD grade, but these did not form a continuous layer. There was, however, continuity between larger vesicles internal to the RPE basement membrane, smaller vesicles traversing the basement membrane, and BLinD external to the basement membrane (e.g., Figure 6C,D). Vesicles further extended into the elastin layer and outer collagenous zone of BrM, generally becoming smaller with increasing distance from the RPE.

## 4. Discussion

While the defining light microscopic lesion of AMD is the presence of continuous sub-macular BLamD, BLinD has, to date, been accepted as a co-occurring lesion observable by electron microscopy [3,4,19]. We found BLinD upon ultrastructural analysis of the RPE-BrM interface in a clinically and histologically validated cohort of adult human eyes *without* AMD. Because none of these eyes had continuous BLamD, our findings suggest that BLinD, when strictly defined, is likely an age-related lesion that precedes the onset of early AMD.

The use of a grading scale for BLinD thickness revealed an association with age, BrM hyalinisation and microscopic RPE abnormalities in this cohort of normal and normal aged eyes. This grading may be helpful in future studies to semi-quantitatively assess if age-related changes in BrM lipid content [6] is attributable to BLinD and whether BLinD thickness corresponds to AMD histological grade. BLinD was first described by Sarks as “an unbroken layer of vesicular membranous debris” external to the RPE basement membrane upon electron microscopic examination [5]. The same study defined undulations within this layer as soft drusen, and this was confirmed by correlation with soft drusen found through in vivo fundoscopy. The first published use of the term “BLinD” to describe this material appeared in 1993 and was defined as “vesicular material present throughout BrM”, with each vesicle measuring “up to 120 nm” in diameter [13]. Our grading scale for BLinD is in accord with these original definitions and extends their utility by providing a semi-quantitative estimate. It may be helpful to clarify that the term ”pre-BLinD”, defined by Chen et al. [19] as a uniform, non-undulating layer ranging from 0.6–1.1 µm in thickness, is equivalent to BLinD Grades 1 to 2 in our study.

Our finding that BLinD thickness is correlated with both age and BrM hyalinisation also agrees with published observations. With ageing, there is a change in BrM matrix composition [20], reflected histologically by progressive degrees of hyalinisation [2,11]. Age-related BrM matrix changes are known to impede bidirectional transport between the RPE and photoreceptors [21,22,23]. These changes slow the transit of macromolecules and their carriers, some of which may manifest as the accumulations of debris within BrM.

In this study, the term “electron-lucent vesicle” is used purely as an ultrastructural descriptor of BLinD composition. In the general biological literature, a “vesicle” is defined as a sac bound by a selectively permeable, bilayered lipid membrane [24]. Extracellular vesicles facilitate intercellular communication and transport a wide array of biomolecules between cells, including proteins, nucleic acids, and lipids. The commonest extracellular vesicles observed in tissue are exosomes (30–100 nm) and microvesicles (100–1000 nm) [25]. While exosomes are derived from the endosomal pathway and microvesicles by directly budding from a cell’s surface, both pathways are involved in physiological and pathological processes. Importantly, the cargo of both types of extracellular vesicles can be rich in lipids [26,27].

Several observations have been used to infer that BLinD is comprised of solid lipoprotein “particles” rather than vesicles. These include (i) the presence of esterified (bound) cholesterol in macular BrM [27], ”basal deposits” and ”drusen” [7]; (ii) the increased electron density of some BLinD constituents seen using lipid-preserving (OTAP) electron microscopy processing [8,28]; (iii) the presence of ApoB and ApoE (lipoproteins) in ”basal deposits” and ”drusen” [7]; and (iv) the appearance of spherical structures within BLinD by quick-freeze deep-etch scanning electron microscopy [29]. This biogenesis framework derives from an extensive range of published literature on the pathology of atheromatous plaques [30], which may be a poor fit to describe senescence-associated changes in bidirectional transport between RPE and the choriocapillaris. In atheroma pathogenesis, blood-derived lipoproteins are forced into an extracellular compartment (the vascular intima) under high arterial blood pressure, made possible once the barrier function of the endothelial monolayer is damaged [31]. This context shares few similarities with the physiological lipid transport that occurs between the RPE (neuroepithelium) and the choriocapillaris (low pressure, fenestrated capillary network) across a specialised extracellular matrix (BrM) within an immune-privileged intraocular microenvironment.

Lipoproteins act as vehicles for the transport of hydrophobic lipids within the aqueous environment of circulating blood; they are not a component of the normal extracellular matrix. Cell-to-cell transport of lipids, including esterified cholesterol and lipoproteins, occurs largely via extracellular vesicles [32,33]. Since BLinD can be found in normal eyes without AMD, its accumulation likely represents a physiological and dynamic process that increases with age and BrM hyalinisation.

Unless lipid transport between the RPE and the choriocapillaris can be uniquely established to occur via the release of free lipoproteins, it is likely that the cholesterol and lipoproteins observed within BrM and BLinD exist as the cargo of extracellular vesicles. This conceptual framework accommodates the key ultrastructural observations of BLinD. The electron-lucent vesicles in BLinD range from 50–200 nm in our study, which accords with previous observations [13]. Thus, they fall more within the range of exosomes and microvesicles (30–1000 nm) rather than lipoproteins (7–90 nm) [25,34]. More critically, BLinD vesicles are highly variable in both shape and size, whereas lipoproteins are uniformly shaped and sized [34]. Using the OTAP method, the most consistent finding is the electron-dense, lipid-rich outer membranes of BLinD structures [8], although there is insufficient evidence to determine if these are monolayer-like lipoproteins [8] or bilayer-like extracellular vesicles [3]. More importantly, OTAP reveals BLinD structures with a range of electron densities, from very dense to entirely lucent, suggesting a highly variable lipid content. This conflicts with findings expected for lipoproteins, which have a consistently high and uniform lipid content. The significance of the larger electron-lucent vesicles we found internal to the RPE basement membrane remains uncertain. However, the basolateral docking of vesicles prior to transport is biologically more plausible than attempting to fit this observation into the atheroma pathogenesis conceptual framework.

Limitations of this study include the unknown and long postmortem time to fixation in some eyes, which may have led to reduced tissue preservation. Furthermore, variation in fixation techniques (glutaraldehyde and formalin fixation) between paraffin and resin sections may have impacted the degree of RPE detachment observed and limited direct comparability. Additionally, thickness measurements may have been impacted by the angle at which the tissues were sectioned.

While our study is morphological, we contend that the science of BLinD biogenesis is far from settled, and that lipid transport mechanisms between the RPE and the choriocapillaris deserve further investigation. The point at which BLinD develops into soft drusen indicates the biological disease threshold for AMD. Evidence to date suggests that this threshold occurs when there is continuous macular BLamD [3]. Our data supports the use of BLinD grading to complement the AMD grading based on BLamD, serving to improve histopathological validation in study cohorts.

## Figures and Tables

**Table 1:** **Cohort characteristics.** Ten normal Group I and 16 normal aged Group II eyes met the inclusion criteria. Compared with Group II eyes, the Group I eyes were significantly younger (*p* = 0.0014, 95% CI 4.8–17.6), with a trend towards better visual acuity (*p* = 0.11, 95% CI –0.05–0.5). The fundus was normal in all eyes at the last in vivo (clinical) examination, except for two eyes with non-specific pigment spots (noted as “vitelliform spots”) seen in the context of a few small hard drusen.

Sarks Group ^a^	Age (Years)	Sex	Post-Mortem Delay (Hours)	Visual Acuity ^b^ (LogMAR)	Follow-Up to Death Interval (Months)	Fundus Appearance Last Examination	Macular Paraffin Sections Reviewed	Macular Semithin Resin Sections Reviewed	Macular TEM Photographs Reviewed
**Group I** **No macular BLamD**									
**1**	73	M	Unknown	0	36	Normal	15	22	49
**2**	58	M	3	0	1	Normal	-	10	12
**3**	80	M	Unknown	0.18	36	Normal	-	4	6
**4**	55	M	Unknown	0	12	Normal	16	4	7
**5**	60	M	Unknown	0	5	Pigment spot	15	3	47
**6**	59	M	5	-	10	Normal	15	5	3
**7**	55	M	1	0	1	Normal	17	12	43
**8**	59	M	Unknown	0.18	10	Normal	20	10	55
**9**	69	M	Unknown	0	8	Normal	-	2	17
**10**	62	M	Unknown	0.18	7	Normal	15	3	17
**Group II** **Patchy macular BLamD**									
**1**	72	M	Unknown	−0.08	6	Normal	-	2	43
**2**	72	M	1.5 h	0	24	Normal	22	2	14
**3**	81	M	2 h	0.18	Unknown	Normal	15	6	55
**4**	74	M	12 h	0.18	2	Normal	-	8	11
**5**	74	M	12 h	0.8	2	Normal	-	7	2
**6**	88	M	Unknown	0.3	4	Normal	19	13	11
**7**	74	F	Unknown	0	9	Normal	-	9	16
**8**	74	F	Unknown	CF	9	Normal	-	1	12
**9**	72	M	Unknown	0.07	9	Normal	-	3	26
**10**	73	M	4.5 h	0.18	36	Normal	-	2	11
**11**	57	M	Unknown	0.3	31	Normal	-	12	30
**12**	66	M	Unknown	0.6	5	Normal	-	2	15
**13**	86	M	Unknown	0.18	5	Normal	18	2	17
**14**	77	M	11 h	0	64	Normal	-	8	2
**15**	77	M	11 h	0	64	Pigment spot	-	5	31
**16**	70	F	Unknown	0.18	1	Normal	15	5	8

^a^ Histopathological grade based on the appearance of macular BLamD. ^b^ Corrected distance VA at last clinical visit. Abbreviations: VA—visual acuity; BLamD—basal laminar deposit; TEM—transmission electron microscopy.

**Table 2:** **BLinD in normal and normal aged eyes by ultrastructural analysis.** BLinD was found in both normal Group I and normal aged Group II eyes. BrM hyalinisation was more advanced in Group II compared with Group I (*p* = 0.013; 95% CI 0.6–5.3), with a positive correlation between the degree of BrM hyalinisation and BLinD thickness (*p* = 0.049; 95% CI 0.05–2.69). The presence of BLinD in TEM photographs was not significantly associated with the RPE detachment seen in paraffin (*p* > 0.99) or resin sections (*p* > 0.99). There was no significant association between the RPE detachment seen in paraffin versus resin sections (*p* = 0.12).

Sarks Group ^a^	Presence of BLinD ^b^	BLinD Thickness (Grade) ^b^	BrM Hyalinisation (Grade)	RPE Morphology	RPE Detachment in Paraffin Section	RPE Detachment in Semithin Resin Section
**Group I** **No macular BLamD**						
**1**	Present	1	2	Preserved	Diffuse	Focal
**2**	None	0	2	Preserved	-	None
**3**	Present	1	2	Preserved	-	Focal
**4**	Present	1	1	Foci of hypertrophy and hyperpigmentation	Diffuse	Focal
**5**	None	0	2	Preserved	Diffuse	Diffuse
**6**	None	0	2	Preserved	Diffuse	Focal
**7**	None	0	1	Preserved	Diffuse	None
**8**	None	0	1	Preserved	Diffuse	Diffuse
**9**	None	0	0	Preserved	-	Focal
**10**	None	0	1	Preserved	None	None
**Group II** **Patchy macular BLamD**						
**1**	Present	2	2	Preserved	-	None
**2**	None	0	2	Preserved	Focal	None
**3**	Present	2	2	Preserved	Diffuse	None
**4**	Present	1	2	Foci of hypo and hyperpigmentation	-	Focal
**5**	Present	1	2	Foci of hypo and hyperpigmentation	-	Focal
**6**	None	0	2	Preserved	Focal	None
**7**	Present	2	2	Abnormal	-	Focal
**8**	Present	2	2	Abnormal	-	Focal
**9**	Present	2	2	Preserved	-	None
**10**	None	0	2	Preserved	-	None
**11**	None	0	2	Preserved	-	None
**12**	None	0	1	Preserved	-	None
**13**	Present	1	3	Foci of hypo and hyperpigmentation	Diffuse	Diffuse
**14**	Present	2	3	Foci of hypopigmentation	-	Focal
**15**	Present	2	3	Foci of hypertrophy and thinning	-	None
**16**	None	0	3	Foci of hypertrophy	Focal	None

^a^ Histopathological grade based on the appearance of macular BLamD. ^b^ Ultrastructural findings from TEM photographs of macular resin sections. - indicates that no paraffin sections were available for review. BLinD grades: 0 = none (less than the thickness of the RPE basement membrane; 0.3 µm); 1 = up to 3 × 0.3 µm; 2 = greater than 3 × 0.3 µm. Abbreviations: BLamD—basal laminar deposit; BLinD—basal linear deposit; BrM—Bruch’s membrane; RPE—retinal pigment epithelium; TEM—transmission electron microscopy.

## Data Availability

The original contributions presented in the study are included in the article; further inquiries can be directed to the corresponding author/s.
